# Comparative efficacy of decontamination methods for laparoscopic equipment: a systematic review and meta-analysis

**DOI:** 10.3389/fcimb.2025.1591478

**Published:** 2025-11-18

**Authors:** Jiajing Li, Shibin Gao, SuBo Zhang, Lei Geng, Yusheng Zhao, Tao Zhang, Lei Zhang, Haiyang Li, Yi Sun, Jinxin Wan

**Affiliations:** 1 Department of Medical Imaging, The Second People’s Hospital of Lianyungang, Lianyungang, China; 2 Department of Medical Imaging, Cancer Hospital of Lianyungang, Lianyungang, China; 3 Department of Medical Imaging, The Second People’s Hospital of Lianyungang Affiliated with Kangda College of Nanjing Medical University, Lianyungang, China; 4 Computer Center, Lianyungang Zhituo Energy Saving Electric Co., Ltd., Lianyungang, China; 5 Department of Information System, Lianyungang 149 Hospital, Lianyungang, Jiangsu, China

**Keywords:** healthcare-associated infections, decontamination methods, medical equipment, comparative efficacy, meta-analysis

## Abstract

**Objective:**

To compare the effectiveness of manual cleaning, alkaline multi-enzyme immersion with ultrasonic cleaning, and automatic reprocessing machines in decontaminating laparoscopes through a systematic review and meta-analysis of randomised controlled trials.

**Methods:**

A comprehensive literature search was conducted across PubMed, Embase, Cochrane Library, Web of Science, Sinomed, CNKI and Wanfang databases from inception to February 2025. Randomised controlled trials comparing different cleaning and disinfection methods for laparoscopes were included. The primary outcome was the qualified rate of decontamination, defined as meeting predetermined thresholds for each detection method (visual cleanliness, protein <6.4 μg/cm², ATP <200 RLU, negative occult blood). Heterogeneity was assessed using I² statistics, with subgroup analyses by detection method and intervention type. Risk of bias was evaluated using the Cochrane risk of bias tool.

**Results:**

Eleven randomised controlled trials involving 4,661 cases were included. Meta-analysis showed that alkaline multi-enzyme immersion with ultrasonic cleaning improved qualified decontamination rates compared with manual cleaning alone when assessed by visual inspection (risk ratio [RR] = 1.07, 95% CI: 1.02–1.13, P < 0.01) and occult blood test (RR = 1.12, 95% CI: 1.02–1.23, P < 0.05). The 7% improvement in first-pass cleaning qualification translates to potentially preventing contamination in approximately 70 additional instruments per 1,000 processed. Automatic reprocessing machines showed similar improvements (RR = 1.08, 95% CI: 1.01–1.16, P < 0.05). Low heterogeneity (I² < 25%) was observed across most outcomes.

**Conclusion:**

The evidence suggests that combined cleaning methods provide modest but clinically meaningful improvements over manual cleaning alone, though certainty is limited by methodological constraints and geographic concentration of studies. Healthcare facilities should consider implementing enhanced protocols while weighing resource availability, training requirements, and local infection prevention priorities.

## 1 Introduction

The cleaning and disinfection of medical equipment, particularly endoscopes and laparoscopes, remain critical components of infection prevention in healthcare settings. Inadequate decontamination of these instruments can lead to biofilm formation, cross-contamination and healthcare-associated infections (HAIs). With the increasing use of minimally invasive procedures, laparoscopes and other endoscopic instruments have become essential in modern surgical practice, requiring effective cleaning and disinfection protocols to ensure patient safety (de Santiago et al., 2022).

The traditional manual cleaning method, although widely used, has limitations, including operator-dependent variability, the potential for human error and difficulties in cleaning complex instrument designs with small lumens and intricate components (Collins, 2021). To overcome these challenges, alternative methods have been developed, such as alkaline multi-enzyme immersion cleaning combined with ultrasonic cleaning and automated reprocessing and disinfection systems (Rutala and Weber, 2021).

Proper decontamination underpins the entire reprocessing procedure for reusable medical devices. Even high-level disinfection or sterilisation may be compromised if instruments are not adequately sanitised beforehand, as organic residues can shield microorganisms from sterilants and disinfectants (Rutala and Weber, 2019). According to established guidelines, thorough cleaning can reduce bioburden by 2–6 log_10_, substantially improving the effectiveness of subsequent disinfection or sterilisation processes (Association for the Advancement of Medical Instrumentation., 2021).

The complex design of laparoscopes presents particular challenges for reprocessing. These instruments often contain small lumens, intricate joints and delicate optical components that can harbour biological debris if not properly processed. Studies have shown that protein residues as low as 6.4 μg/cm² can interfere with sterilisation efficacy, underlining the critical importance of thorough cleaning before disinfection or sterilisation (Eichel et al., 2021).

Various detection methods have been employed to assess cleaning efficacy, including visual inspection, magnification with a light source, protein residue testing, occult blood testing, adenosine triphosphate (ATP) bioluminescence and specialised cleaning verification tools. Each method has distinct advantages and limitations in terms of sensitivity, specificity, ease of use and cost-effectiveness (Heuvelmans et al., 2021). Professional organisations recommend using multiple verification methods to ensure cleaning adequacy, as no single test can detect all potential contaminants (Speer et al., 2023).

Despite international guidelines emphasising validated cleaning methods and quality control measures, significant gaps remain in our understanding of comparative cleaning efficacy (Walsh, 2024). Current evidence is limited by heterogeneity in cleaning protocols between studies, lack of standardised outcome definitions, absence of long-term clinical outcome data linking cleaning adequacy to infection rates, and limited data from diverse geographic regions and healthcare settings. Furthermore, existing systematic reviews have not quantitatively synthesised the available randomised controlled trial evidence, leaving clinicians without clear guidance on optimal cleaning strategies.

To provide high-quality evidence to inform clinical practice, we conducted a systematic review and meta-analysis of randomised controlled trials to quantitatively compare the efficacy of different decontamination methods for laparoscopes. The primary objective was to determine whether enhanced cleaning methods (alkaline multi-enzyme with ultrasonic cleaning or automated reprocessing) provide superior decontamination compared with manual cleaning alone. Secondary objectives included evaluating the reliability of various detection methods for assessing cleaning adequacy and identifying optimal protocols for clinical implementation.

## 2 Materials and methods

### 2.1 Protocol registration and reporting

This systematic review and meta-analysis was conducted according to the Preferred Reporting Items for Systematic Reviews and Meta-Analyses (PRISMA) 2020 guidelines. The protocol was registered at https://inplasy.com/. The PRISMA checklist is provided in **Supplementary File 1**.

### 2.2 Literature search strategy

A systematic literature search was conducted from database inception to February 2025. Multiple electronic databases were searched, including PubMed (n = 12), Embase (n = 3), Cochrane Library (n = 6), Web of Science (n = 4), Sinomed (n = 2), CNKI (n = 378) and Wanfang (n = 165). The search used combinations of the following keywords: ‘cleaning and disinfection’, ‘cleaning and disinfection method’, ‘randomised controlled trial’, ‘medical equipment’, ‘medical device’ and ‘endoscope’. Boolean operators (AND, OR) were applied to combine search terms as appropriate.

The final PubMed search syntax was as follows: ‘(medical device[Title/Abstract] OR endoscope*[Title/Abstract] OR laparoscope*[Title/Abstract]) AND (clean* OR decontaminat* OR disinfect*) AND (randomised controlled trial[Publication Type])’.

Similar Boolean logic with appropriate field tags and truncation symbols was applied across all databases. No language restrictions were applied. Reference lists of relevant reviews and included studies were manually screened. Grey literature and trial registries were not systematically searched, representing a potential source of publication bias.

### 2.3 Inclusion and exclusion criteria

Studies were selected based on predefined inclusion and exclusion criteria. To be eligible, studies had to be randomised controlled trials focusing on laparoscopes, comparing different cleaning and disinfection methods and reporting the qualified rate of cleaning as assessed by at least one detection method.

The inclusion criteria were as follows: (1) randomised controlled trial design; (2) study participants specifically focusing on laparoscopes; (3) interventions clearly comparing different cleaning and disinfection methods; (4) outcome measures reporting the qualified cleaning rate evaluated by at least one detection method; (5) full-text availability for complete evaluation.

Studies were excluded under the following conditions: (1) non-randomised controlled trial design; (2) duplicate publications or studies with overlapping data; (3) incomplete data or unclear methodology; (4) reviews, case reports or conference abstracts without primary data.

The “qualified rate” was defined as the proportion of instruments meeting predetermined cleanliness thresholds for each detection method: visual inspection (no visible soil), protein residue (<6.4 μg/cm²), ATP bioluminescence (<200 relative light units), occult blood test (negative result), magnifying glass inspection (no visible residue at 5× magnification), and 3M cleaning test rod (pass according to manufacturer specifications).

### 2.4 Literature screening and data extraction

Two independent reviewers screened titles and abstracts, then assessed full texts against inclusion criteria. The inter-rater reliability was measured using Cohen’s kappa coefficient (κ = 0.82, indicating substantial agreement). Disagreements were resolved through discussion or third-reviewer consultation.

Data extraction used a standardised, pilot-tested form capturing: study characteristics (author, year, country, design); sample characteristics (size, equipment type); intervention details (cleaning methods, protocols, duration, concentrations); outcome measures (detection methods, qualified rates, definitions); and methodological quality indicators.

For missing data or unclear reporting, study authors were contacted for clarification. When authors could not be reached or data remained unavailable, studies were included but the missing elements were noted in the risk of bias assessment. All extracted data were cross-checked by a second reviewer.

### 2.5 Quality assessment

The methodological quality of included studies was assessed using the Cochrane risk of bias tool, evaluating seven domains: (1) random sequence generation; (2) allocation concealment; (3) blinding of participants and personnel; (4) blinding of outcome assessment; (5) incomplete outcome data; (6) selective reporting; (7) other bias. For each domain, studies were categorised as having low risk, unclear risk or high risk of bias.

### 2.6 Statistical analysis

Meta-analysis was conducted using Review Manager 5.4 software. Risk ratios (RR) with 95% confidence intervals were calculated for dichotomous outcomes. Heterogeneity was evaluated using the chi-square test and I² statistic (25%, 50%, and 75% indicating low, moderate, and high heterogeneity, respectively). Fixed-effects models (Mantel–Haenszel) were used when I² < 50%; random-effects models (DerSimonian and Laird) when I² ≥ 50%.

Subgroup analyses were performed by detection method and intervention comparison. Sensitivity analyses were conducted by excluding studies with high risk of bias in key domains. Publication bias was assessed visually using funnel plots and statistically using Egger’s test when ≥10 studies were available per outcome. Statistical significance was set at P < 0.05.

## 3 Results

### 3.1 Literature screening process

**Figure 1:**
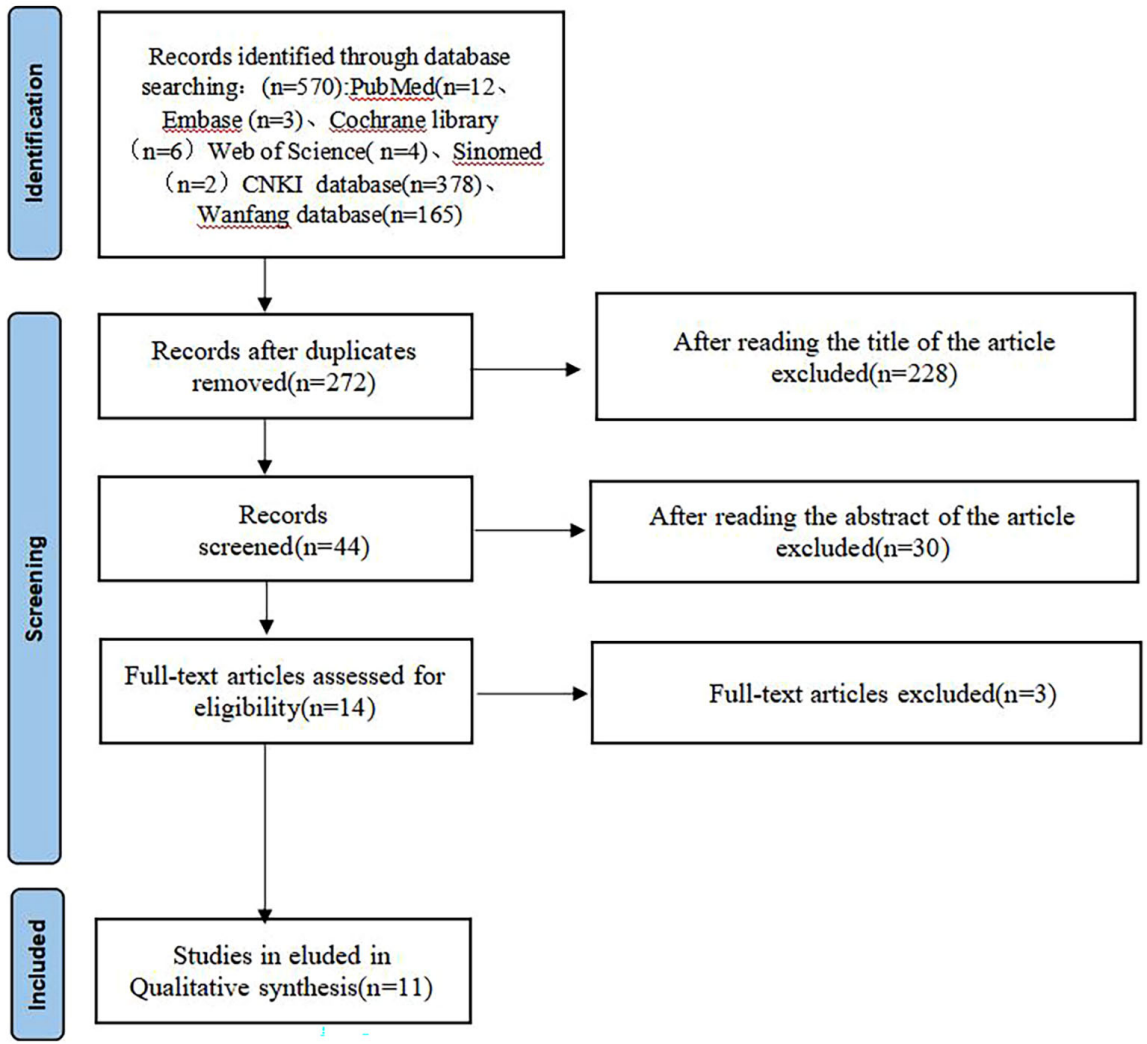
The flow chart of literature screening.

The initial database search yielded 570 records. After removing 272 duplicates, 298 records remained for screening. Following title and abstract screening, 254 were excluded. Full-text assessment of 44 articles led to exclusion of 33 studies (non-randomised design n = 12, wrong intervention n = 8, wrong outcome n = 7, duplicate data n = 3, incomplete data n = 3). Finally, 11 randomised controlled trials met all inclusion criteria. The literature screening process is shown in **Figure 1**.

### 3.2 Basic characteristics of included studies

Eleven randomised controlled trials involving 4,661 cases (2,339 experimental, 2,322 control) were included. All studies focused on laparoscopes, with sample sizes ranging from 140 to 800 cases. Publication years ranged from 2016 to 2025. All studies were conducted in China, limiting geographic generalisability.

The interventions compared were: (1) manual cleaning (control); (2) alkaline multi-enzyme immersion cleaning + ultrasonic cleaning; (3) automatic cleaning and disinfection machine. Detection methods varied across studies, with visual inspection being most common (11 studies), followed by ATP bioluminescence (4 studies) and occult blood testing (4 studies). Study characteristics are summarised in **Table 1**.

**Table 1:** Basic characteristics of included literature.

Author (year)	Research object	Type of study	Number of cases (E/C)	Intervention measure	Outcome index
(Halmans et al., 2024)	laparoscope	randomised controlled trial	300/300	a,c	①,③,⑤
(Xiao and Tang, 2023)	laparoscope	randomised controlled trial	250/250	a,b	①,⑤
(Yan, 2025)	laparoscope	randomised controlled trial	270/253	a,b	①,⑤
(Deng and Lu, 2018)	laparoscope	randomised controlled trial	70/70	a,b	①,⑤
(Sun, 2021)	laparoscope	randomised controlled trial	167/167	a,b	①
(Hu et al., 2021)	laparoscope	randomised controlled trial	400/400	a,c	①,②
(Gou, 2021)	laparoscope	randomised controlled trial	100/100	a,c	①,②
(Kang, 2020)	laparoscope	randomised controlled trial	132/132	a,b	①,③,④
(Wang et al., 2023)	laparoscope	randomised controlled trial	250/250	a,b	①,③,④
(Ren et al., 2024)	laparoscope	randomised controlled trial	250/250	a,b	①,④,⑥
(Jiang et al., 2016)	laparoscope	randomised controlled trial	150/150	a,b,c	①,④,⑥

a: manual cleaning, b: alkaline multi-enzyme immersion cleaning + ultrasonic cleaning, c: Automatic cleaning and disinfection machine cleaning and disinfection ①: visual method, ②: 5x magnifying glass with light source Detection methods, ③: residual protein, ④: occult blood test, ⑤: ATP bioluminescence rapid detection, ⑥: 3M cleaning test rod test.

### 3.3 Quality assessment of included studies

**Figure 2:**
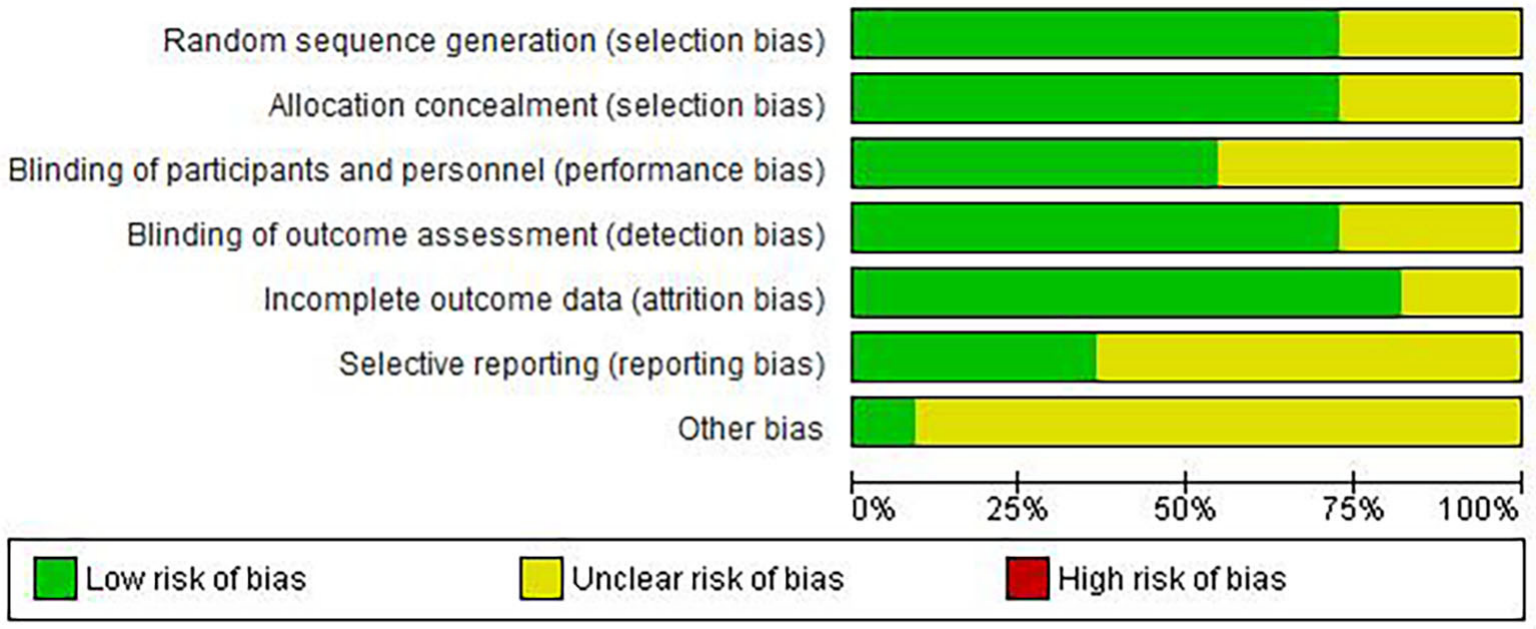
Risk of bias graph: review authors’ judgements about each risk of bias item presented as percentages across all included studies.

**Figure 3:**
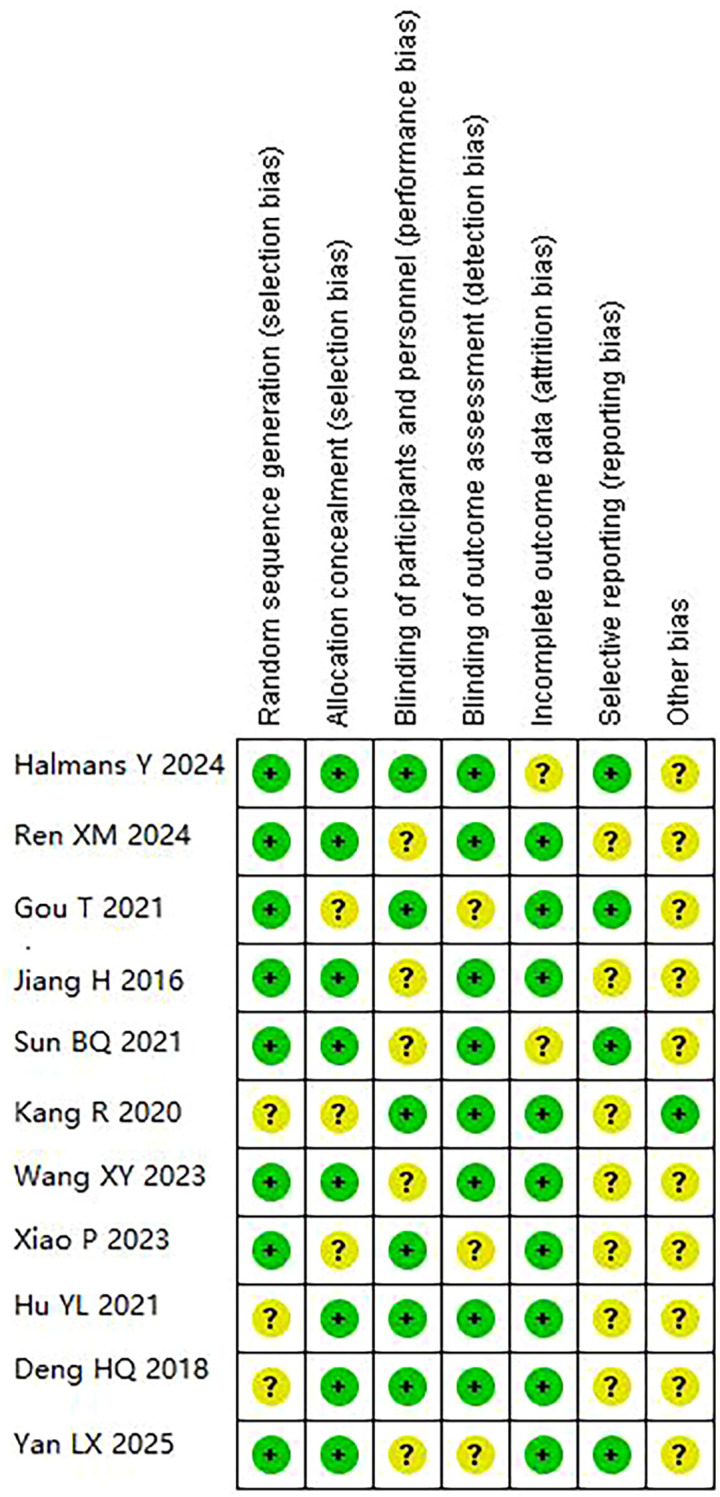
Risk of bias summary: review authors’ judgements about each risk of bias item for each included study. Green circles indicate low risk of bias; yellow question marks indicate unclear risk of bias.

Risk of bias assessment revealed moderate overall quality (**Figures 2**-**3**). Adequate random sequence generation and allocation concealment were reported in 72.7% of studies. Blinding of participants/personnel was achieved in 54.5% of studies, outcome assessment blinding in 72.7%. Complete outcome data were available in 81.8% of studies. Selective reporting bias was low in 36.4% of studies, unclear in 63.6%. Other bias remained unclear in 81.8% of studies, potentially affecting result interpretation.

### 3.4 Meta-analysis results

#### 3.4.1 Visual inspection method

**Figure 4:**
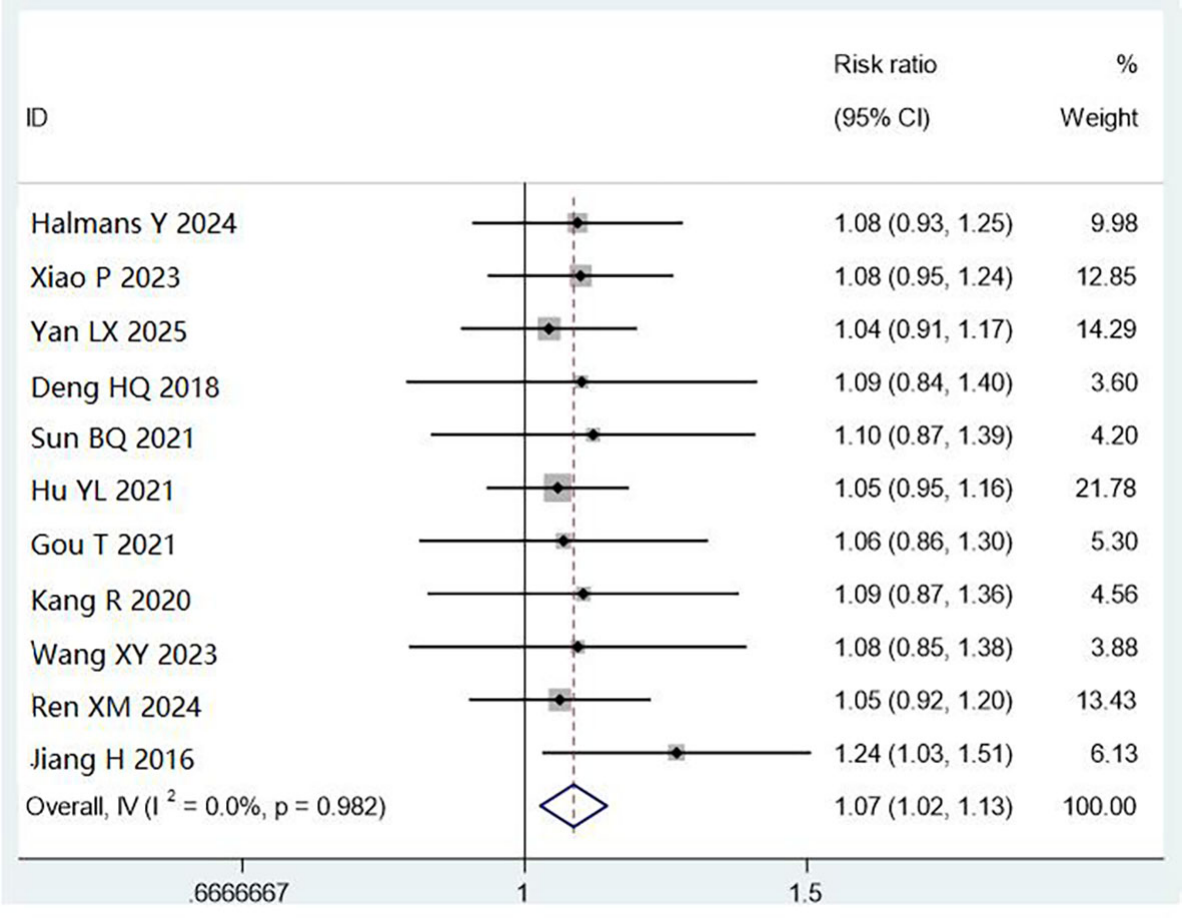
Meta analysis results of comparison of qualified rate of cleaning by visual method.

All 11 studies (4,661 cases) reported cleaning qualification by visual inspection. Combined cleaning methods improved qualification rates compared with manual cleaning (RR = 1.07, 95% CI: 1.02–1.13, P < 0.01), with low heterogeneity (I² = 0%). This 7% improvement translates to approximately 70 additional instruments passing inspection per 1,000 processed, potentially reducing contamination risk. However, the clinical significance depends on baseline infection rates and reprocessing volumes at individual facilities (**Figure 4**).

Subgroup analysis showed both alkaline multi-enzyme + ultrasonic cleaning (RR = 1.06, 95% CI: 1.01–1.12, P < 0.05) and automatic reprocessing (RR = 1.08, 95% CI: 1.01–1.16, P < 0.05) were superior to manual cleaning alone.

#### 3.4.2 Occult blood test

**Figure 5:**
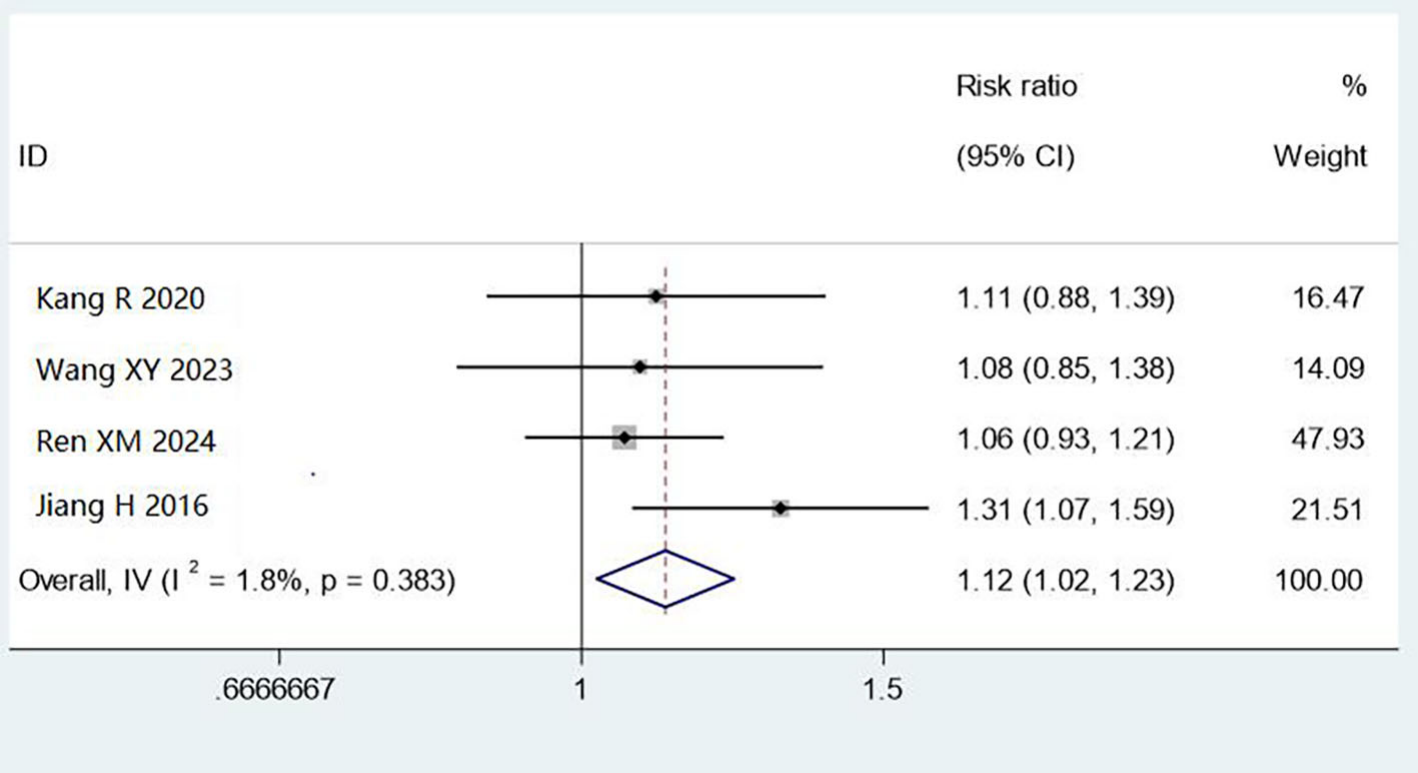
Meta analysis results of comparison of qualified rate of cleaning by occult blood test.

Four studies (1,564 cases) assessed cleaning by occult blood testing. Combined methods improved qualification rates compared with manual cleaning (RR = 1.12, 95% CI: 1.02–1.23, P < 0.05), with low heterogeneity (I² = 1.8%). The 12% improvement represents clinically meaningful enhancement in removing blood proteins, critical for preventing prion transmission and ensuring sterilisation efficacy (**Figure 5**).

#### 3.4.3 Adenosine triphosphate bioluminescence detection

**Figure 6:**
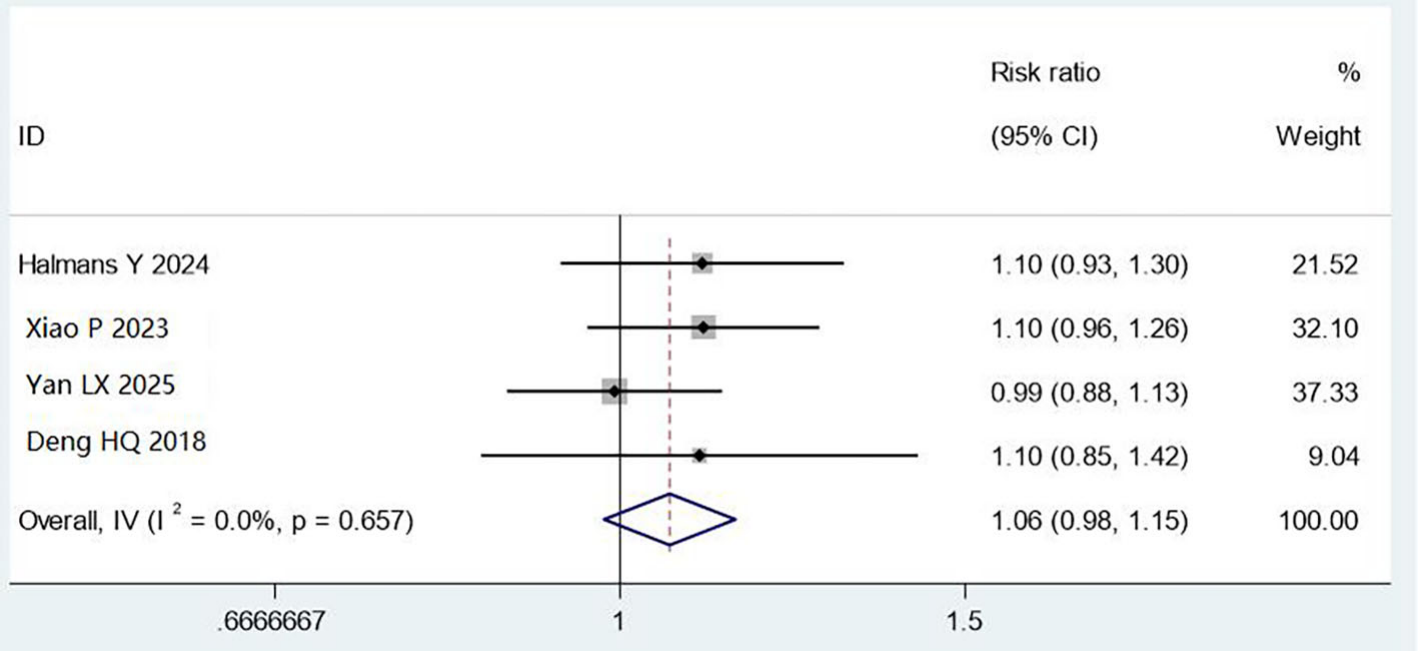
Meta analysis results of comparison of qualified rate of cleaning by ATP bioluminescence test.

Four studies (1,763 cases) used ATP bioluminescence testing. While combined methods showed a trend toward improvement (RR = 1.06, 95% CI: 0.98–1.15), this did not reach statistical significance (P = 0.657), with no heterogeneity (I² = 0%). The lack of significance may reflect high baseline cleaning success rates, ATP measurement variability, or interference from cleaning agents. Further research with standardised ATP thresholds is needed (**Figure 6**).

#### 3.4.4 The 3M cleaning test rod test

**Figure 7:**
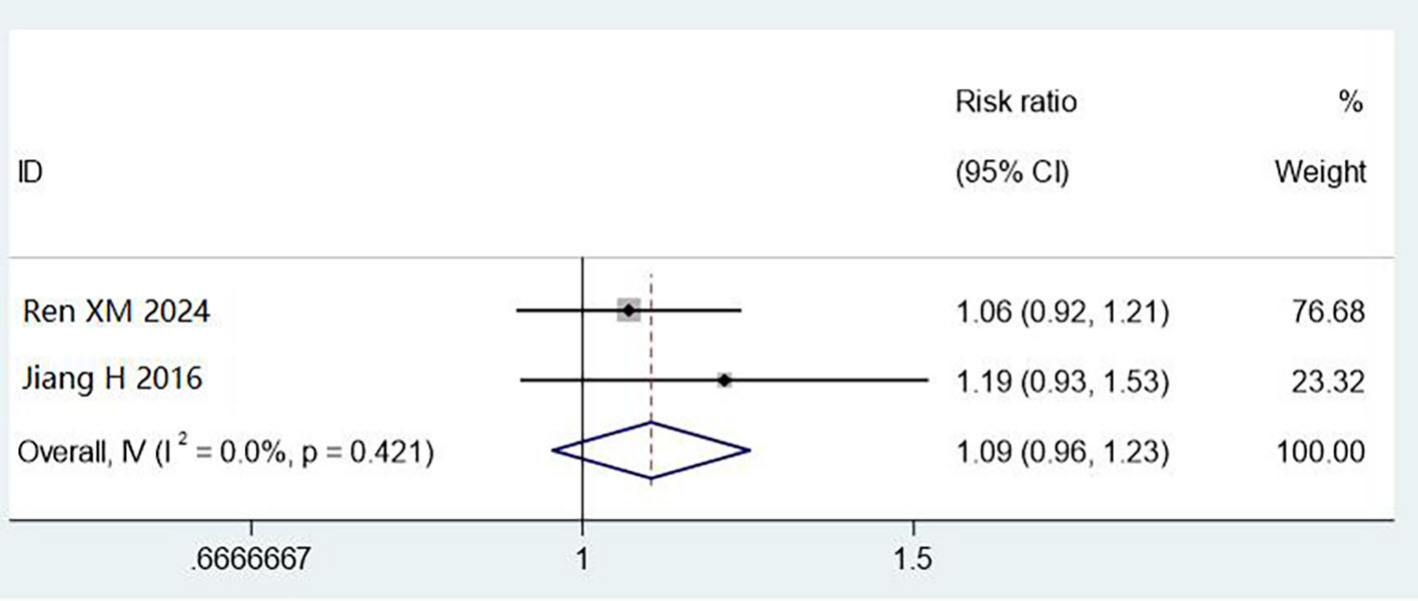
Meta analysis results of comparison of qualified rate of cleaning by 3M cleaning test rod test.

Two studies (800 cases) assessed cleaning using 3M test rods. Combined methods showed non-significant improvement (RR = 1.09, 95% CI: 0.96–1.23, P = 0.421), with no heterogeneity (I² = 0%). The wide confidence intervals and limited sample size preclude definitive conclusions. The observed 9% improvement warrants investigation in larger studies (**Figure 7**).

#### 3.4.5 5× magnifying glass with light source

**Figure 8:**
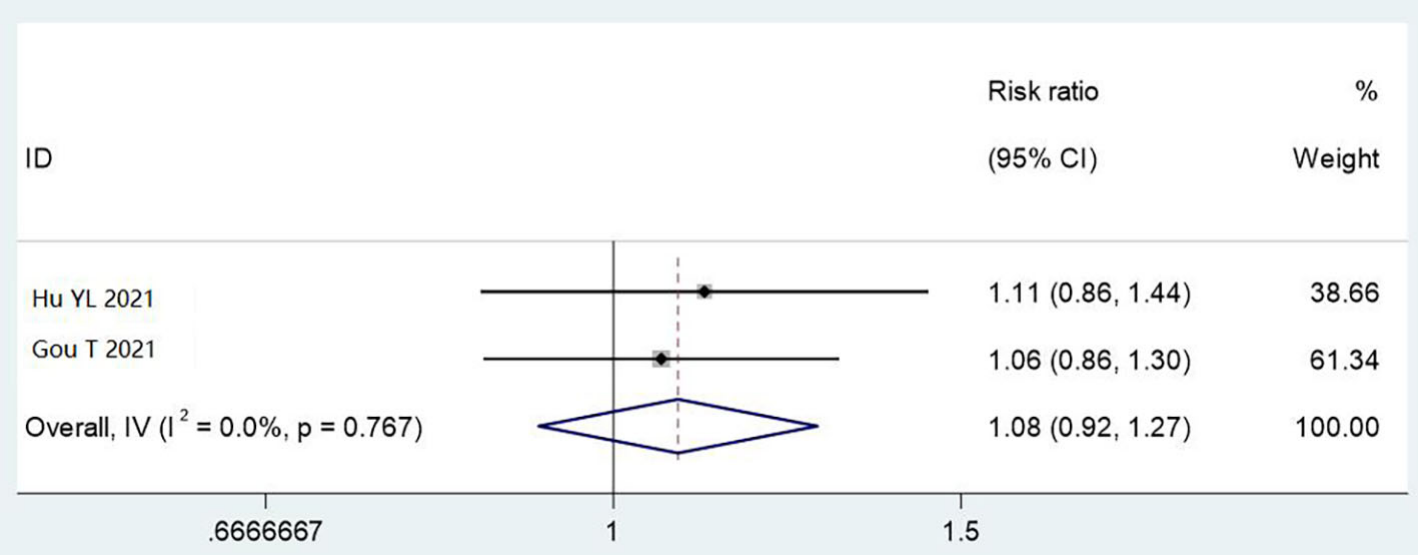
Meta analysis results of comparison of qualified rate of cleaning by 5x magnifying glass with light source detection methods.

Two studies (1,000 cases) used magnified inspection. Automatic reprocessing showed non-significant improvement over manual cleaning (RR = 1.08, 95% CI: 0.92–1.27, P = 0.767), with no heterogeneity (I² = 0%). Limited data prevent firm conclusions about this detection method’s utility (**Figure 8**).

#### 3.4.6 Publication bias assessment

Funnel plot analysis for visual inspection outcomes (11 studies) showed slight asymmetry suggesting potential publication bias favouring positive results. Egger’s test approached significance (P = 0.08), indicating possible small-study effects. Sensitivity analysis excluding smaller studies (n < 200) did not substantially alter the main findings (RR = 1.06, 95% CI: 1.01–1.11).

## 4 Discussion

### 4.1 Interpretation of main findings

Our findings suggest that combined cleaning methods, particularly alkaline multi-enzyme immersion cleaning with ultrasonic cleaning, achieve modest but clinically meaningful improvements over manual cleaning alone for laparoscope decontamination. The evidence supports a 7-12% improvement in cleaning qualification rates across different detection methods, though the certainty of evidence remains moderate due to methodological constraints. The 7% improvement observed with visual inspection methods represents approximately 70 additional instruments passing inspection per 1,000 processed. In high-volume facilities processing 100 laparoscopes daily, this could prevent 7 potentially contaminated instruments from entering the disinfection/sterilisation cycle each day. While this improvement is statistically significant, its clinical relevance depends on baseline infection rates and the specific risk profile of the patient population served. The variation in effect sizes across detection methods provides insights into the nature of cleaning improvements. The 12% improvement with occult blood testing suggests that enzymatic cleaning may be particularly effective at removing blood proteins, which are critical contaminants for prion transmission and can shield microorganisms from sterilants. The non-significant findings for ATP bioluminescence (6% improvement) may reflect measurement variability or interference from cleaning agents rather than lack of efficacy.

### 4.2 Clinical and operational implications

The practical meaning of a 6-12% improvement in cleaning adequacy requires careful consideration of implementation contexts. For infection prevention teams in high-resource settings with established quality systems, adopting alkaline multi-enzyme plus ultrasonic protocols could be justified, particularly for high-risk procedures or immunocompromised patient populations. According to established guidelines, a comprehensive cleaning protocol for complex surgical instruments should include immediate pre-cleaning at the point of use, thorough manual cleaning with appropriate detergents, and the use of mechanical assistance such as ultrasonic cleaning when indicated (Stucky et al., 2024). Our findings provide evidence supporting these recommendations. However, in resource-limited settings, the modest improvements must be weighed against competing priorities. Basic improvements in manual cleaning training, compliance monitoring, and point-of-use cleaning may yield greater returns than advanced technologies. Investment in automated cleaning and disinfection systems may be justified by the potential for improved standardisation and reduced labour requirements, particularly in high-volume settings. However, proper validation and regular monitoring of these systems remain essential to ensure consistent performance (Rutala et al., 2023).For perioperative nursing practice, our findings have important implications. Clinical nurse specialists play a central role in quality assurance and safety management, particularly during challenging periods such as the COVID-19 pandemic, when enhanced protocols for instrument reprocessing became critical. Studies have identified knowledge gaps among reprocessing personnel regarding best practices for cleaning and disinfection (Taunk et al., 2022). Comprehensive education on the principles of cleaning, appropriate use of enzymatic detergents, operation of ultrasonic cleaners, and implementation of quality monitoring procedures should be provided to all staff involved in instrument reprocessing. The non-significant findings for certain detection methods (ATP bioluminescence, 3M test rod) should not be interpreted as evidence against enhanced cleaning. Rather, these results highlight the complexity of measuring cleaning efficacy and the need for multiple assessment methods. Current recommendations suggest using objective methods to verify cleaning effectiveness rather than relying solely on visual inspection (Benowitz et al., 2020).

### 4.3 Comparison with previous studies

Our findings align with and extend the broader literature on medical device reprocessing. Previous prospective studies evaluating endoscope reprocessing in real-world settings have found that manual cleaning alone is often insufficient to remove all biological debris (Day et al., 2021). Research has emphasised the importance of standardised protocols and mechanical cleaning assistance to achieve consistent outcomes. Similarly, studies have demonstrated that automated cleaning systems provide more reliable removal of bioburden from flexible endoscopes compared with manual cleaning (Lee et al., 2021). Automated systems have been shown to consistently achieve a >99.9% reduction in protein, haemoglobin and bioburden, whereas manual cleaning results remain more variable. These findings support the advantages of automated and enhanced cleaning methods demonstrated by our meta-analysis. The efficacy of enzyme-based cleaners is also well documented. Studies investigating the cleaning performance of different enzymatic detergents on artificial biofilm have shown that multi-enzyme formulations are substantially more effective than single-enzyme products or non-enzymatic detergents (Tian et al., 2021). These results reinforce the suitability of alkaline multi-enzyme solutions for laparoscope cleaning. Ultrasonic cleaning has been recognised as a valuable adjunct to chemical cleaning methods. Evaluations of the synergistic effects of enzymatic detergents and ultrasonic energy have demonstrated that the combination provides superior removal of protein-based soils compared with either method alone (Tessarolo et al., 2022). The cavitation effect of ultrasonic cleaning is particularly effective in removing contaminants from complex instrument designs with small lumens and crevices, as commonly found in laparoscopic instruments.

### 4.4 Mechanism of different cleaning methods

While understanding mechanisms supports our findings, the clinical implications remain paramount. The superior efficacy of combined cleaning methods can be attributed to their complementary mechanisms of action. Manual cleaning relies solely on mechanical action and is highly dependent on the operator’s technique and thoroughness. Studies have demonstrated substantial variability in cleaning effectiveness when procedures were performed by different personnel, even when the same protocol was followed (Weber et al., 2023).

Enzyme-based cleaners operate through specific biochemical mechanisms. Different enzymes target distinct biological residues: proteases break down proteins, lipases degrade fatty substances, amylases dissolve starches and cellulases break down cellulose-based materials (Yang et al., 2023). Multi-enzyme formulations provide comprehensive coverage across the diverse types of biological debris commonly present on surgical instruments. Ultrasonic cleaning enhances mechanical removal by means of acoustic cavitation. Ultrasonic waves passing through a liquid medium create microscopic bubbles that implode on contact with surfaces, generating localised high pressure and temperature to dislodge contaminants (Tan et al., 2022). This mechanism is particularly effective for accessing areas that are difficult to reach by manual brushing. Automated cleaning and disinfection machines offer standardised processes with controlled parameters. Studies evaluating the reproducibility of automated endoscope reprocessors have shown that these systems provide consistent cleaning results across multiple cycles, with minimal operator-dependent variability (Casini et al., 2021).

### 4.5 Detection methods for cleaning efficacy

Our meta-analysis evaluated several detection methods for assessing cleaning efficacy, each with distinct advantages and limitations. Visual inspection, although widely used in practice, has notable limitations. Studies have shown that it cannot reliably detect protein residues below 200 μg, which is well above the threshold that can interfere with sterilisation (Akinbobola et al., 2021). The meta-analysis indicated that when more sensitive detection methods were applied, the advantages of enhanced cleaning methods became clearer.

Adenosine triphosphate bioluminescence testing provides a rapid quantitative assessment of biological residues. This method detects ATP, present in all living cells, and offers a measure of biological contamination (Hu et al., 2024). However, it cannot differentiate between viable microorganisms and organic debris, and results may be affected by certain detergents and disinfectants. Protein detection methods, including occult blood tests, offer specific identification of protein residues. Sensitivity evaluations have shown that these methods can detect protein levels as low as 1 μg, making them valuable tools for verifying cleaning efficacy (Nixon, 2024). The meta-analysis showed substantial advantages of enhanced cleaning methods when assessed using occult blood tests. Commercial tools such as the 3M Clean-Trace ATP system provide standardised approaches to detecting residual contamination. These systems have been found to be reliable indicators of cleaning adequacy when properly validated and used in accordance with manufacturers’ instructions (Marchese et al., 2021).

### 4.6 Methodological considerations and certainty of evidence

The moderate certainty of our evidence reflects several methodological considerations that may have influenced findings. The inability to blind cleaning personnel introduces potential performance bias, though objective outcome assessment partially mitigates this concern. The unclear risk of other bias in 81.8% of studies, reflecting incomplete reporting of conflicts of interest and protocol deviations, warrants cautious interpretation.

The geographic concentration of all 11 studies in China represents our most significant limitation for generalisability. Healthcare systems vary substantially in infrastructure, water quality (mineral content, pH, temperature), detergent formulations, and baseline practices. Without representation from other regions, we cannot definitively establish whether these findings translate globally. The potential for regional publication practices and language bias may also affect the representativeness of included evidence.

Publication bias assessment revealed slight funnel plot asymmetry and near-significant Egger’s test (P = 0.08), suggesting possible small-study effects favouring positive results. While sensitivity analysis excluding smaller studies did not substantially alter findings, the possibility of unpublished negative studies cannot be excluded.

### 4.7 Future research priorities

Based on the limitations identified in our analysis, we recommend five priority areas for future research, each directly addressing current evidence gaps:

#### 4.7.1 Multicentre international trials

To address geographic limitations, we strongly recommend establishing an international consortium for endoscope reprocessing research. Standardised approaches to measuring and reporting cleaning efficacy are needed. Variability in testing methods and thresholds for acceptable cleanliness complicates comparisons across studies (Lee et al., 2020).

#### 4.7.2 Clinical outcome linkage

Research linking cleaning efficacy to clinical outcomes such as surgical site infection rates would strengthen the rationale for adopting enhanced cleaning protocols. Longitudinal studies tracking both cleaning quality metrics and patient outcomes could provide valuable insights.

#### 4.7.3 Economic evaluation

Comprehensive cost-effectiveness analyses are essential. Considerations beyond direct costs should include staff time, processing capacity, instrument longevity and potential healthcare savings from reduced infections (Pontes et al., 2023).

#### 4.7.4 Innovation assessment

Investigation into newer technologies for instrument cleaning and disinfection would expand the evidence base. Emerging approaches such as electrolysed water, cold atmospheric plasma and hydrogen peroxide vapour require rigorous evaluation.

#### 4.7.5 Implementation science studies

Research examining how to optimise protocol adherence, staff training, and quality systems for sustainable improvement is critical across different healthcare contexts.

### 4.8 Strengths and limitations

This meta-analysis has several strengths. First, it included only randomised controlled trials, which provide the highest level of evidence for comparing interventions. Second, multiple detection methods were evaluated, offering a comprehensive assessment of cleaning efficacy. Third, the included studies demonstrated moderate to high methodological quality in key domains. Fourth, heterogeneity among studies was low for most outcomes, suggesting consistency of results. However, several limitations temper our conclusions. The geographic concentration in China substantially limits global generalisability. The relatively small number of studies for some detection methods limits statistical power. Heterogeneity in specific protocols within broad cleaning categories may have influenced results. The moderate risk of bias across several domains requires cautious interpretation of the magnitude of effects observed.

## 5 Conclusion

This systematic review and meta-analysis provides moderate-certainty evidence that combined cleaning methods achieve modest improvements in laparoscope decontamination compared with manual cleaning alone. The 7-12% improvement in cleaning qualification rates may translate to meaningful infection prevention benefits, particularly in high-volume settings. However, certainty is limited by methodological constraints, geographic concentration of studies, and absence of clinical outcome data.

Healthcare facilities should consider implementing enhanced cleaning protocols based on local infection rates, resources, and quality improvement priorities. Successful implementation requires not only equipment acquisition but also comprehensive training, standardised protocols, and robust quality monitoring systems. Institutions must weigh the modest benefits against implementation costs and competing infection prevention priorities.

Future research should prioritise international collaboration, standardised outcome assessment, and linkage to clinical endpoints to strengthen the evidence base for endoscope reprocessing standards. Until more definitive evidence emerges, facilities should focus on optimising whichever cleaning method they employ through rigorous training, protocol adherence, and continuous quality improvement.

## Data Availability

The original contributions presented in the study are included in the article/**supplementary material**. Further inquiries can be directed to the corresponding authors.
